# Dissecting inflammation in the immunemetabolomic era

**DOI:** 10.1007/s00018-025-05715-8

**Published:** 2025-04-28

**Authors:** Patricia P. Ogger, Peter J. Murray

**Affiliations:** Immunoregulation Research Group, Max Planck Institute of Biochemistry, Martinsried, 82152 Germany

**Keywords:** Metabolomics, Efferocytosis, IL-10, Kynurenine, Spatial resolution, Trained immunity, Chronic inflammatory disease

## Abstract

The role of immune metabolism, specific metabolites and cell-intrinsic and -extrinsic metabolic states across the time course of an inflammatory response are emerging knowledge. Targeted and untargeted metabolomic analysis is essential to understand how immune cells adapt their metabolic program throughout an immune response. In addition, metabolomic analysis can aid to identify pathophysiological patterns in inflammatory disease. Here, we discuss new metabolomic findings within the transition from inflammation to resolution, focusing on three key programs of immunity: Efferocytosis, IL-10 signaling and trained immunity. Particularly the tryptophan-derived metabolite kynurenine was identified as essential for efferocytosis and inflammation resolution as well as a potential biomarker in diverse inflammatory conditions. In summary, metabolomic analysis and integration with transcriptomic and proteomic data, high resolution imaging and spatial information is key to unravel metabolic drivers and dependencies during inflammation and progression to tissue-repair.

## Info box ‘definition of metabolomics’

The term **‘Metabolome’** was first used in 1998 to describe all metabolites in an organism, where metabolites are defined as small-molecular-weight molecules of < 1500 Dalton [1]. **Metabolomics** is therefore the analysis (identification and quantification) of all metabolites in an organism, tissue, cell or system [2]. Historically, studying metabolites was limited to **metabolic profiling** and **metabolic fingerprinting/footprinting** [3]. For metabolic profiling, a defined group of metabolites is analyzed, usually from a specific pathway i.e., glycolysis or the tricarboxylic acid (TCA) cycle. Metabolic fingerprinting aims to identify a set of metabolites to define a sample, while for metabolic footprinting only secreted, extracellular metabolites are considered. More recently, metabolomics encompasses **targeted** and **untargeted metabolomics**. Targeted metabolomics analyzes a predefined set of metabolites and is strictly speaking not an ‘omics’ approach. Recent development of mass spectrometric and bioinformatic tools have greatly streamlined the efficiency and accuracy of untargeted metabolomics, which attempts to capture information about all (known) metabolites in a sample. Since metabolites are heterogenous in nature (i.e. hydrophilic, hydrophobic, positive or negatively charged), extraction and acquisition methods determine the scope of the analysis. Thus, to perform global untargeted metabolomics, different extraction and acquisition approaches have to be combined. A subset of metabolomics is **lipidomics**, which identifies and quantifies only lipid molecular species [4, 5]. Targeted and untargeted metabolomics face different challenges: while targeted metabolomics is limited by the number of pre-defined metabolites and ideal reference standards, no reference standards are used in untargeted metabolomics, and missing masses in reference libraries make it hard to identify unknown metabolites. Furthermore, untargeted metabolomics relies on combination of different extraction methods to identify diverse metabolite classes, which may introduce bias. New metabolomic resources that help to overcome these challenges include the **Human Metabolome Database** (HMDB5.0) containing > 220,000 metabolites for reference information [6] and the metaboanalyst platform (**MetaboAnalyst 6.0**), which allows spectra processing and integration with transcriptomic data and provides functional insights [7, 8].

## Info box ‘metabolomic techniques’

Both targeted and untargeted metabolomics can be **quantitative**, hence determining actual concentrations of metabolites, or **semi-quantitative**, only measuring relative intensities. Nuclear magnetic resonance **(NMR)** spectroscopy was the first analytical platform for metabolite analysis and is still being used as such for its advantageous minimal sample preparation, exceptional reproducibility and ability to identify highly polar compounds [9]. Still, mass spectrometry-based analytical platforms are more specific and sensitive because they measure more metabolites at lower concentrations. Mass spectrometry-based metabolomic analysis requires initial separation techniques such as gas or liquid chromatography. With gas chromatography-mass spectrometry **(GC-MS)**, volatile compounds can be analyzed immediately, while polar metabolites such as amino acids, amines and carboxylic acids need to be derivatized first, resulting in time consuming sample preparation [10, 11]. Liquid chromatography-mass spectrometry (**LC-MS**) offers analysis of the largest range of metabolite classes and polarities, for which different acquisition modes and separation techniques need to be combined [12]. Several acquisition methods are used for untargeted metabolomics: full scan, data independent analysis (**DIA**) and data dependent analysis (**DDA**). Both DIA and DDA enable higher spectral information and hence larger range of identified metabolites. However, using real-time analysis of MS-data, DDA produces the highest number of identifiable metabolites at the expense of acquiring data at lower speed and potentially introducing bias into the analysis [13]. In addition to these traditional techniques, recent advances allow spatial and single cell metabolomics, and even subcellular resolution such as single organelle metabolomics [14, 15]. Table 1 provides an overview of novel spatial- and single-cell metabolomic techniques. **Spatial metabolomics**, based on mass spectrometry imaging (MSI), combines spatial sample information with local desorption and ionization, followed by mass analysis. Matrix assisted laser desorption/ionization (**MALDI**) is most versatile and commonly used, while desorption electrospray ionization (**DESI**) is faster due to no required sample preparation, but secondary ion mass spectrometry (**SIMS**) has the highest spatial resolution [16, 17]. **SpaceM** combines MALDI-MSI with light microscopy to enable single cell metabolomics of cultured cells [18] and has recently been upgraded to also enable isotope tracing at spatial single cell resolution (^**13**^**C-SpaceM**) [19]. For detailed discussion of mass spectrometry-based metabolomic techniques and analysis methods, their history, advantages and disadvantages as well as sample preparation, we refer to separate expert reviews [3, 13, 20, 21].

**Table 1:** Overview of imaging, spatial and single-cell metabolomics techniques

Abbreviation	Technique	Advantages	Ref.
metaFISH	MALDI-MSI combined with fluorescent in situ hybridization (FISH)	Links spatial metabolome to fluorescent imaging	[22]
3D OrbiSIMS	SIMS-MSI in 3D with Orbitrap mass analyzer	Combines high spatial resolution of SIMS with high mass resolving power of Orbitrap; Label-free	[23]
SIMS/MALDI MSI	Multimodal Mass spectrometry imaging combining MALDI with SIMS and secondary electron imaging	Complementary imaging information; High resolution imaging in all three modes without sample transfer	[24]
3D-SMF	3D spatially resolved metabolic profiling framework	Incorporates metabolic profiles with 3D cell specificity via isotope tagged antibody library	[25]
SEAM	Spatial single nuclear metabolomics	Algorithm for single nuclear segmentation in combination with SIMS; Label free	[14]
BCA	Biomolecular component analysis of organelle lipidomics	Raman microscopy-based lipid mapping combined with fluorescent organelle labelling	[15]
SpaceM / Multiplex Immunofluorescent MSI	MSI combined with light microscopy or multispectral imaging	In situ single cell metabolome, linked to imaging cues	[18, 26]
^13^C-SpaceM	Spatial single cell isotope tracing	Combines ^13^C-isotope tracing with fluorescent microscopy; pre-and post-MALDI microscopy	[19]
scSpaMet	Single cell spatially resolved metabolic framework	Combines untargeted spatial metabolomics with targeted multiplexed protein imaging	[27]

TOF– time of flight; MALDI– matrix-assisted laser desorption/ionization; SIMS– secondary ion mass spectrometry

## Introduction

Immunometabolism encompasses the broad penumbra of metabolic dependencies driving immune cell functions and kinetic changes that occur in metabolism during an immune response [28, 29]. The latter is a vital part of understanding how an initial inflammatory response changes in direction. For example, during wound healing and tissue repair which can either restore tissue integrity or lead to the perpetuation of inflammation and fibrosis. Most chronic diseases are defective at this decision point [30]. The role of immune metabolism, specific metabolites and cell-intrinsic and -extrinsic metabolic states across the time course of any immune-inflammatory response are emerging knowledge, as novel metabolomic approaches (see box ‘metabolomic techniques’ and Table 1) can identify unexpected and unexplored metabolite changes. This information can be linked to high resolution imaging and new spatially-resolved techniques to better understand how the cells of the immune system adapt their metabolism throughout an immune response.

What are the roles of these metabolites and metabolic pathways? What is the physiology of kinetic changes in metabolism during inflammation and which metabolic changes are cause or consequence of pathophysiology? What is the purpose of re-wiring metabolic pathways at different stages of an immune response? Emerging metabolomic approaches provide direct information on biochemical function and are colloquially deemed ‘the best omics’ by some to answer these questions [31]. In addition to information obtained from targeted metabolic profiling, fingerprinting and isotope tracing, metabolic flux analysis and expression of metabolite transporters, untargeted metabolomics can identify previously unconnected metabolic pathways in immune cells. It yields high potential for identifying metabolic pathways suitable for therapeutic manipulation, especially when correlated with spatial tissue information or integrated with transcriptomic and proteomic data.

Over the last decade, specific metabolites including succinate [32], itaconate [33, 34], lactate [35] and kynurenine [36], amino acids (i.e. glutamine [37], arginine [38], tryptophan [39]) as well as fatty acid biosynthesis and oxidation [40, 41] have been shown to exert direct control over specific immune cell responses; in other words, these metabolites are signaling molecules. Via immune metabolic signaling, metabolites support a spectrum of cellular functions during immune activation in addition to cytokine and chemokine signaling. In terms of metabolic re-wiring, the degree of the immune metabolic shifts, dictated by transcriptomic and proteomic changes, reflects the type and duration of the immune response. Metabolomic analysis can aid to identify pathophysiological patterns in disease; in essence, how immune cells change their metabolism across a response is a “fingerprint” of the type of response and may be used as biomarker of disease state and, potentially, prognosis.

While the endpoint of acute inflammation is ultimately tissue repair, deregulated immune responses result in tissue damage and chronic inflammation. Upon an insult, innate immune cells issue the first response, in parallel to local stromal cells: Neutrophils, tissue macrophages and dendritic cells (DCs) produce pro-inflammatory chemokines and cytokines [42, 43] while, respectively, neutralizing and presenting antigens. During the ensuing amplification phase, lymphocytes (including T cells, B cells, innate lymphoid cells and natural killer cells) are recruited, leading to further cytokine and growth factor release, co-stimulation and antibody production, while infiltrating monocytes differentiate into tissue macrophages in a tissue- and context-dependent manner [44, 45]. By passing checkpoints, the inflammation progresses towards the termination phase [46], culminating in wound healing and corpse removal facilitated by macrophages (efferocytosis) [47, 48]. Immune metabolic orchestration, via cell-intrinsic metabolic shifts and cross-talk via metabolite [49, 50], amino acid [51] and lipid signaling [52, 53], contributes to and supports each phase of inflammation functionally [54–59]. Nevertheless, our understanding of the metabolic changes that occur in parallel to the immune response and implications for specific cellular functions (i.e. phagocytosis vs. efferocytosis; cytokine and antibody production) are limited for the moment.

Here, we review new metabolomic findings within the transition from inflammation to resolution, which sets the course for either repair or chronic non-resolving inflammation. This review does not cover the remarkable advances in understanding metabolomic changes linked to cancer cell biology or the metabolic intersection of immunity with cancers [60], as experts have ably covered the most recent research in this area [61–68]. Nor do we address the vast contribution of microbial and endogenous microbiome metabolites to immune system function. Furthermore, the SARS-CoV-2 pandemic in the ‘Omics-Era’ has generated unprecedented numbers of studies covering immune metabolic changes during SARS-CoV-2 infection and COVID-19 specific metabolomic biomarkers, which exceed the scope of this review and have been discussed elsewhere [69–74]. Instead, we briefly discuss new findings about metabolic pathways underlying three key programs of immunity, which define the intersection between inflammation and resolution: Efferocytosis, IL-10 signaling and trained immunity. Additionally, we discuss new frontiers of metabolomic advances (spatial-, imaging-, and single cell metabolomics) and multi-OMIC integration (i.e. with deep visual proteomics and spatial transcriptomics), which provides opportunity to identify therapeutic targets and biomarkers of inflammatory diseases.

## Metabolic drivers of efferocytosis

Efferocytosis is the process by which phagocytes remove apoptotic corpses (AC) from all tissues. Cell turnover under homeostatic conditions is immense as ~ 1% of all cells die each day and need to be rapidly removed [75]. Corpse identification via “find me” and “eat me” signals is well understood [76, 77] as is the fact that corpse elimination and digestion is a vital step to prevent inflammation since the exposure of nucleic acids, peroxidated lipids and other debris from dead cells can drive further inflammation and eventually autoimmunity [78]. Furthermore, efferocytosis contributes to the transition from inflammation to resolution by inducing production of pro-repair mediators including prostaglandin E_2_ (PGE_2_), TGF-β1 and IL-10, while reducing expression of pro-inflammatory mediators nitric oxide (NO), TNF-α, IL-1β and IL-6 [79–81]. What is less understood about efferocytosis is the process by which phagocytes that engulf and digest cell corpses subsequently re-program their transcriptional and metabolic responses to express genes and proteins that support the transition to a tissue repair phenotype. This process is an emerging area of research for several reasons: First, the precise scope and function of the tissue repair phenotype is not fully understood. Second, cytokines and other signaling molecules can further promote or inhibit efferocytosis-associated gene expression. Third, cellular corpse origin elicits different phagocyte responses [82]. Unsurprisingly, efferocytosis triggers broad metabolic changes in the phagocyte [48, 82, 83]. Substantial energy is required to process a corpse to its constituent base molecules (i.e. nucleic acids, lipids, amino acids and carbohydrates), hence efferocytosis is dependent on glucose uptake and functional glycolysis, while inducing transcription of glycolytic pathway intermediates as shown in an in vitro efferocytosis assay using LR73-hamster phagocytes and apoptotic Jurkat T cells [84]. A central question in understanding efferocytosis centers on the connection between the digestion process and the metabolic changes in the phagocyte that follow, and how these are linked to transcriptional changes. To understand metabolic reactions associated with efferocytosis, we need to consider (a) metabolites and metabolic programs in myeloid cells required for efferocytosis, (b) metabolites acquired from apoptotic target cells and (c) metabolites that are secreted during or after efferocytosis, acting as signaling metabolites. Thus, the application of targeted and untargeted metabolomics is a key tool in understanding efferocytosis. Key questions include: Are building blocks such as metabolites, amino acids and lipids derived from apoptotic target cells taken up by efferocytosing myeloid cells? How are they transported from the phagolysosome into the cytosol and membranes? And how does efferocytosis and corpse obliteration drive the functional phenotype of efferocytosing myeloid cells– that is progression from inflammation towards resolution?

### The problem of transport of AC-derived metabolic building blocks

**Fig. 1:**
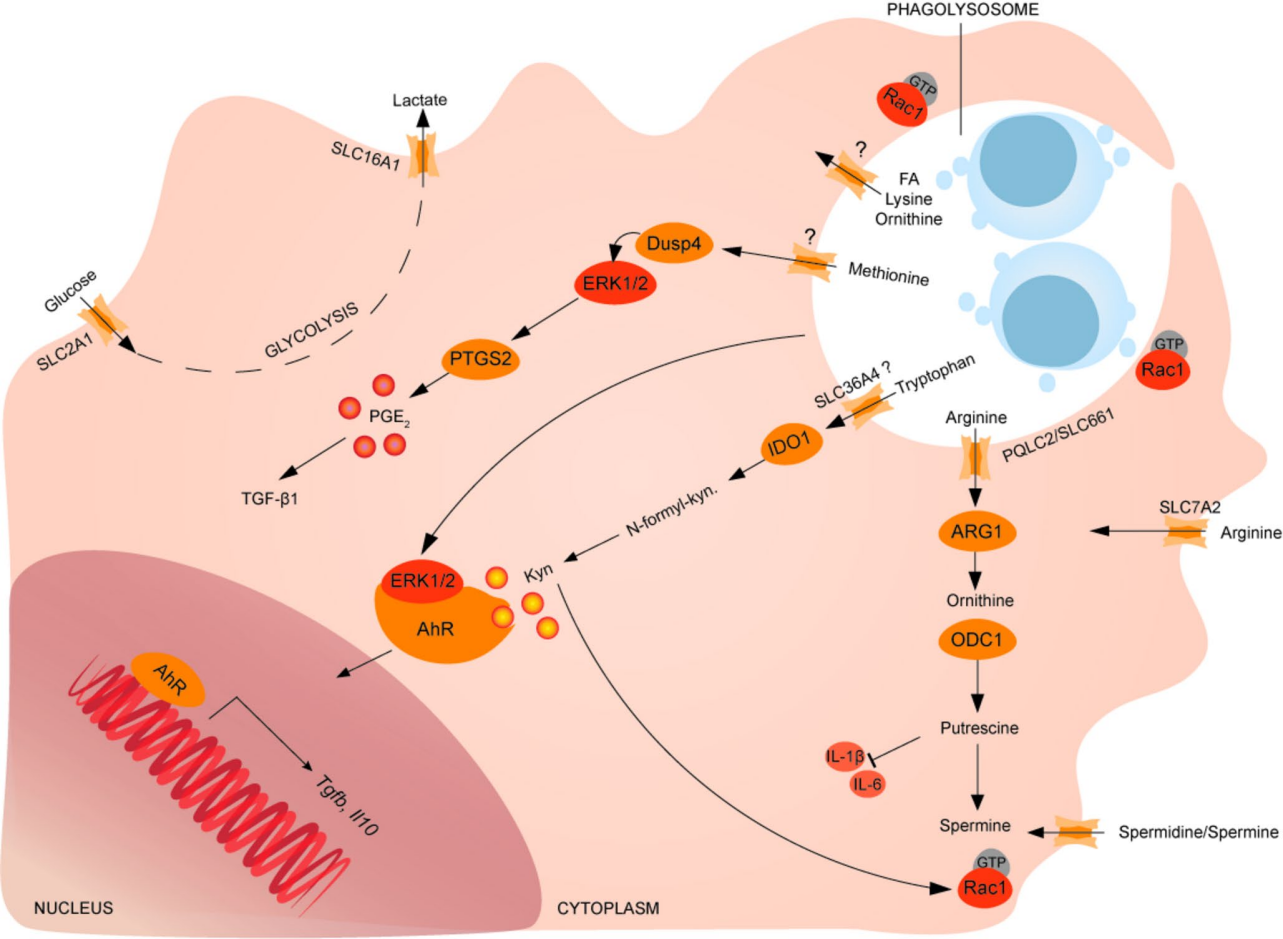
Novel aspects involved in the metabolic control of efferocytosis

Upon processing the apoptotic corpse in the phagolysosome, recyclable materials including amino acids are exported into the cytosol. At the same time, efferocytosing phagocytes alter their cell surface expression of many amino acid transporters [84], as shown on LR73 hamster phagocytes. Therefore, a balance between corpse-derived amino acids, intracellular amino acids reservoirs and external amino acid supplies must be struck. Conceivably, amino acid homeostasis during corpse obliteration is dynamic and affects phagocyte amino acid homeostasis. So far, our understanding of the dynamics of this process is a challenge that can be met with new tools of immunometabolomics. While there have been many recent advances in understanding the solute carrier (SLC) amino acid transporters on the cell surface, the mechanisms that control amino acid exit from the lysosome are poorly understood [48]. Arginine for example, is transported across the plasma membrane via SLC7A2 [85] and has recently been suggested to be exported from the lysosome of efferocytosing bone marrow-derived macrophages (BMDMs) by the cationic amino acid transporter Pqlc2/SLC66A1 [86]. Initially identified as lysosome transporter for lysine and arginine (LAAT-1) in *C. elegans* [87], silencing of *Pqlc2* in murine macrophages resulted in impaired arginine-putrescine metabolism and dysfunctional continuous efferocytosis. These data suggest Pqlc2 for lysosomal arginine transport in murine macrophages [86], which will need to be confirmed in human myeloid cells. Furthermore, increased arginine within the lysosome is sensed by the lysosomal neutral amino acid transporter SLC38A9, which signals to mTORC1 [88, 89] and regulates lysosomal efflux of essential amino acids [90]. The transport of other amino acids such as lysine and methionine, or indeed fatty acids recycled from the corpse out of the phagolysosome is less well understood. The transporter SLC36A4 had been suggested to transport tryptophan, however only in *Xenopus laevis* oocytes [91], until Sukka et al., recently showed colocalization of SLC36A4 with apoptotic corpse containing phagolysosomes and increased SLC36A4 expression upon AC engulfment in BMDMs [92]. RNA-sequencing of efferocytosing LR73 hamster phagocytes identified altered expression of 33 SLCs, with increased expression of those related to carbohydrate metabolism [84]. Particularly SLC2A1 (GLUT-1) and SLC16A1 are necessary to support efferocytosis via glucose uptake and lactate export, respectively (Fig. 1). While SLC expression is distinct between resting, efferocytosing and phagocytosing macrophages in vitro [84], spatial- and organelle-proteomics and metabolomics from in vitro assays as well as verification in human tissue samples will be necessary to resolve the complexity of SLC expression and differentiate between transport across the plasma membrane and in and out of the phagolysosome.

### Efferocytosis rewires amino acid metabolism pathways

Untargeted LC-MS of LPS-primed peritoneal macrophages fed with either apoptotic, necrotic or IgG coated Jurkat cells ex vivo, showed that upon engulfment of any target cell, amino acid content increases, including methionine, arginine and lysine [80]. This was not the case upon engulfment of non-cell targets, suggesting that these amino acids are target cell derived. Although it is unclear how apoptotic corpse-derived methionine is exported from the phagolysosome into the cytosol, it is needed for methylation of the ERK1/2 phosphatase Dusp4. ERK1/2 in turn activates the prostaglandin-endoperoxide synthase 2 (Ptgs2), leading to PGE_2_ synthesis and subsequent TGF-β1 production [81]. Using targeted LC-MS analysis of amino acids and acylcarnitines, increased arginine and ornithine levels were confirmed in BMDMs upon AC engulfment in vitro [86]. Furthermore, McCubbrey et al., identified a distinct polyamine signature in macrophages upon engulfment of apoptotic but not necrotic or IgG coated target cells in vitro [80]. Using isotope labelled arginine and fluorescent-tagged spermine, they found that the highly increased arginine upon AC engulfment (in vitro) does not contribute to de-novo polyamine synthesis, but rather that polyamine import from the environment is increased upon efferocytosis [80]. These results are in contrast to an earlier study, which reported that putrescine synthesis via ornithine decarboxylase 1 (Odc1) was dependent on the aforementioned lysosomal arginine transporter Pqlc2, indicating putrescine synthesis from apoptotic corpse-derived as opposed to environmental arginine [86]. While this study used IL-4 treated BMDMs, McCubbrey et al. investigated AC engulfment in LPS-activated macrophages, which may explain the observed differences in de-novo polyamine synthesis from arginine due to distinct arginase 1 (Arg1) expression in each system [93]. In both scenarios, putrescine is required for continuous efferocytosis and polyamine metabolism contributes to the immunosuppressive effect of efferocytosis (Fig. 1). Overall, amino acid metabolism is rewired in efferocytosing macrophages to re-cycle apoptotic corpse-derived methionine, arginine and ornithine, which enables an immunosuppressive program that includes limiting nitric oxide (NO), TNF-α, IL-1β and IL-6 and inducing the expression of PGE_2,_ TGF-β1 and IL-10 [79–81].

### Efferocytosis and tryptophan metabolism

Targeted analysis of the tryptophan pathway in BMDMs upon engulfment of apoptotic corpses in vitro identified increased tryptophan as well as its metabolites *N*-formyl-kynurenine and kynurenine [92]. Deficiency or inhibition of indoleamine 2,3-dioxygenase 1 (IDO1), which catalyzes the conversion of tryptophan into kynurenine metabolites, limits macrophage efferocytosis capacity and this was rescued by addition of exogenous kynurenine [92]. Indeed, kynurenine, produced from apoptotic cell derived tryptophan [39] activates the aryl hydrocarbon receptor (AhR) [94] and in combination with ERK as secondary signal induces expression of TGF-β and IL-10, mediating immunosuppression [92, 95]. Mechanistically, the Rac family small GTPase 1 (Rac1) bridges the polyamine and kynurenine pathways: Polyamine import from the environment relies on functional Rac1 [80] and Rac1 activation is dependent on the GTP-exchange factor Dbl (*Mcf2*), which is regulated by the Arg1 pathway [86]. This has now been connected to the kynurenine pathway by showing that the efferocytosis-induced Arg1-Dbl-Rac1 axis requires IDO1 and kynurenine [92], Fig. 1.

In many cancer types as well as tumor-infiltrating myeloid cells IDO1/kynurenine is increased [96, 97], which supports cancer cell survival: Tryptophan-derived substrates support de-novo NAD^+^ synthesis in tumor cells and IDO1 restricts tryptophan availability to immune cells in the tumor microenvironment (TME). Furthermore, kynurenine acts as a ligand for the AhR, an immune modulator in the TME, which has also been linked to tumor cell migration [98] and confers resistance to oxidative stress in tumor cells via suppression of ferroptosis, an oxidative-stress induced cell death by lipid peroxidation of cellular membranes [36]. The importance of the tryptophan pathway, increased expression of IDO1/kynurenine and activation of the AhR has also been identified in macrophages infected with Mycobacterium Tuberculosis (Mtb) in vitro. Here, AhR activation via kynurenine lead to inhibition of the JAK/STAT1 pathway and limited T cell recruitment and both AhR and IDO1 have been suggested as potential therapeutic targets for Tuberculosis [99–101]. Indeed, recent studies on Mtb-infected rhesus macaques showed better control of Mtb when administering the IDO1-inihibitor D-1 methyl tryptophan (D1MT) in combination with chemotherapy [102] or in combination with combinatorial anti-retroviral (cART) therapy during co-infection of Mtb and simian immunodeficiency virus (SIV) [103] and human clinical trials are warranted. Less is known about the mechanistic role of kynurenine in inflammatory disease and tissue repair, despite it being one of the most altered metabolites in systemic inflammatory diseases identified by untargeted metabolomic analysis (discussed below) [104–107]. Many open questions on the role of kynurenine in efferocytosis and tissue repair remain: Is apoptotic corpse-derived kynurenine secreted by efferocytosing cells? Does it support extracellular signaling functions? Is AC-derived tryptophan exported and does it induce expression of the extracellular L-amino acid oxidase IL4i1, metabolizing tryptophan in parallel to IDO1 [108]?

### Efferocytosis: a frontier of immunometabolism

In addition to rewired amino acid metabolism, including methionine, arginine, ornithine and tryptophan, lactate secretion upon efferocytosis induces proliferation of pro-resolving macrophages. They manage the progression from inflammation to resolution via Myc protein stabilization [109] and provide a mechanism of immunometabolic connection between AC removal and macrophage-driven tissue repair. Most of the experiments discussed here used murine bone marrow-derived or peritoneal macrophages, which are transcriptionally and metabolically [110, 111] distinct. Human activated macrophages produce less nitric oxide (NO) and maintain functional electron transport upon activation [112–114], as well as relying less on arginine and itaconate for regulation of inflammatory functions [115–118]. It will therefore be essential to translate metabolic underpinnings of efferocytosis to human macrophages during resolution of inflammation. In vivo, IDO1 and the Arg1/Odc1 axis are essential for efferocytosis-dependent resolution of dexamethasone induced thymic inflammation as well as efferocytosis-driven regression of atherosclerosis in *Ldlr*^*−/−*^ mice [86, 92]. Further open questions include the exact role of SLC36A4 and other SLCs in transporting amino acids and fatty acids out of the phagolysosome. Spatial metabolomic analysis will help to illuminate metabolite origin and trafficking upon efferocytosis.

**Fig. 2:**
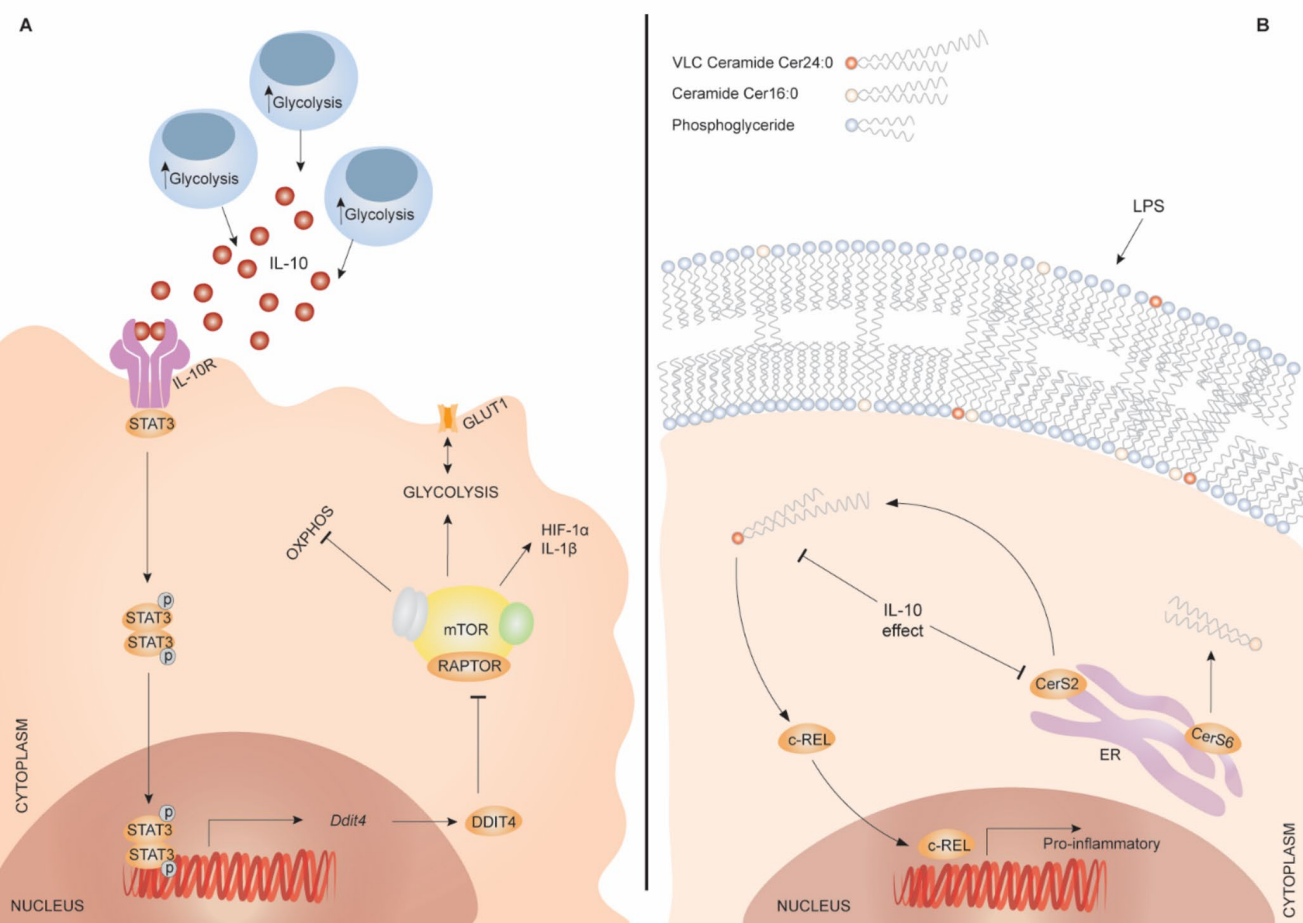
IL-10 induced metabolic reprogramming

During efferocytosis, import of environmental amino acids and glucose import/glycolysis is increased to sustain energy demands. Apoptotic cell derived methionine, arginine and tryptophan are recycled and support continuous efferocytosis and immunosuppression. Methionine is necessary to methylate ERK1/2 phosphatase Dusp4, inducing prostaglandin-endoperoxide synthase 2 (PTGS2) activation and Prostaglandin (PGE_2_) production, ultimately supporting expression of transforming growth factor beta 1 (TGF-β1). Tryptophan is converted into kynurenine (Kyn) via indoleamine 2,3-dioxygenase (IDO1), which activates the aryl hydrocarbon receptor (AhR) in combination with secondary signal ERK1/2, leading to transcription of *Tgfb* and *Il10*. Arginine is converted into putrescine and downstream polyamines via arginase 1 (ARG1) and ornithine decarboxylase 1 (ODC1), which inhibits IL-6 and IL-1β and supports continuous efferocytosis. FA: fatty acid (Fig. 2).

## Metabolic reprogramming and IL-10

IL-10 is an anti-inflammatory cytokine produced during the progression from inflammation to resolution and is essential for steady-state immune regulation. IL-10 is secreted by T cells as functionally dominant source (and additionally by B cells, and activated myeloid cells) and predominantly targets activated myeloid cells, which express the highest amounts of the IL-10 receptor. The signals that lead to IL-10 production in different inflammatory and homeostatic conditions are extraordinarily complex [119, 120]. Similarly, the mechanistic basis of anti-inflammatory IL-10-STAT3 signaling involves complex, gene-specific effects on inflammatory transcription and has been covered in other summaries [121–123]. However, an unexpected effect of IL-10 signaling is on metabolic pathways that regulate mitochondrial metabolism and function. Thus, IL-10 seems to additionally target central energy metabolism as part of its anti-inflammatory effects. So far, two metabolic programs are linked to IL-10 signaling: the oxidative phosphorylation (OXPHOS)/glycolysis balance [124], and fatty acid synthesis [125]. Because IL-10 is an essential cytokine, understanding how metabolism is controlled in parallel to suppression of inflammatory cytokines and chemokines may have valuable clues for translation approaches in inflammatory bowel disease and other chronic conditions where there is not enough IL-10 or IL-10 signaling.

### Metabolic reprogramming supporting IL-10 production

Myeloid cells activated by microbial products shift their central metabolism toward glycolysis. Glycolysis is necessary for IL-10 production via an indirect pathway: Glycolysis-derived ATP drives IL-10 production via conversion into adenosine and activation of the adenosine receptor A2a (A2aR) [126]. This mechanism can be increased in LPS-activated macrophages with the small synthetic molecule TEPP-46. two recent studies highlight the necessity of a functional mitochondrial electron transport chain (ETC), particularly complex III, for IL-10 production in LPS-activated macrophages [127, 128]. It will be intriguing to find out if these pathways are equally important in IL-10 producing lymphoid cells, in humans and in the context of inflammation resolution and tissue repair.

### Metabolic implications downstream of IL-10

Via the IL-10 receptor, IL-10 inhibits glucose uptake by inhibiting SLC2A1/GLUT-1 translocation and instead promotes mitochondrial OXPHOS [124]. IL-10 induces the DNA-damage inducible transcript 4 protein (DDIT4) via activation of STAT3, the central and obligatory transcription factor downstream of the IL-10 receptor (IL-10R). DDIT4 (also known as REDD1) negatively modulates the key metabolic modulator mechanistic target of rapamycin (mTOR) complex 1 (mTORC1) [124]. This leads to reduced glycolysis and reprogramming of macrophage metabolism to support immunosuppressive functions (i.e. downregulation of hypoxia inducible factor 1α (HIF1α), succinate and IL-1β and induction of OXPHOS) along with the induction of mitophagy, clearing dysfunctional and reactive oxygen species (ROS) producing mitochondria [124], Figure 2A. In mice, this metabolic shift induced by IL-10 is, in part, dependent on mitochondrial arginase 2 (Arg2) [129], although the exact mechanisms that connects Arg2 to IL-10 signaling to mitochondria is unclear. Taken together with ~ 30 years of research into IL-10’s anti-inflammatory effects, it seems that a coincident pathway to cytokine and chemokine suppression controls mitochondrial fitness. Since we know that activated myeloid cells sacrifice OXPHOS for glycolysis, perhaps IL-10 balances this effect to sustain the ability of myeloid cells to kill pathogens, consume debris or other key events in the inflammatory trajectory. We note that myeloid cells such as macrophages that developed from the blood monocyte pool and survive in an inflammatory niche will transition to an OXPHOS-dependent phenotype involved in tissue repair: perhaps IL-10 is an exogenous actor in this process, long thought to be a cell intrinsic program? Nevertheless, the IL-10-metabolism connection is ripe for a detailed investigation by OMICs approaches and especially untargeted metabolomics across the time kinetic where IL-10 is known to work.

### IL-10 and sphingolipid metabolism

IL-10 reduces sphingolipid synthesis in activated macrophages [125]. Sphingolipids are part of the plasma membrane, but also function as bioactive lipids by contributing to cellular signaling [130]. Very long chain (VLC) ceramides (one type of sphingolipid), such as Cer24:0 and Cer22:0 induce inflammatory gene expression when added exogenously to activated macrophages, as opposed to long chain ceramides (Cer16:0). VLC ceramides accrue in IL-10-deficient BMDMs, in line with high expression of ceramide synthase 2 (CerS2) [125], which mediates VLC ceramide synthesis [131]. Thus, IL-10 suppresses the production of an endogenous inflammation-driving lipid class. While the mechanism by which IL-10 limits CerS2 activity remains unclear, increased saturated VLC ceramide production has been linked to reduced de-novo synthesis of unsaturated fatty acids. Indeed, restoration of this balance by addition of mono-unsaturated fatty acids (i.e. oleic acid 18:1) limits saturated VLC production [125]. The associated pro-inflammatory gene expression stimulated by saturated VLC ceramides is induced via activation of the transcription factor c-REL, which therefore acts opposing to IL-10 by increasing expression of IL-10-suppressed genes, Figure 2B.

Loss of IL-10 leads to the development of spontaneous inflammation such as inflammatory bowel disease (IBD) or similar colonic inflammation in mice [132]. The new insights into the relationship between IL-10 and lipid metabolism point towards novel targets for the treatment of inflammatory bowel disease and possibly beyond: Inhibition of CerS2 and targeting REL could limit ceramide production and exogenous addition of mono-unsaturated fatty acids might circumvent IL-10 deficiency and push macrophages towards an immunosuppressive and resolving phenotype.

Remaining questions in addition to those revolving around the relationship between IL-10, AhR and kynurenine involve firstly the feasibility of translation of the proposed relationship between IL-10 and negative regulation of glycolysis and ceramide metabolism beyond BMDMs. Secondly, not only macrophages but also T-cell subsets (Th2, Th17 and Treg) produce IL-10 [133] and metabolic requirements for IL-10 production in these remain unresolved. Thirdly, in addition to its anti-inflammatory roles, IL-10 can facilitate chronic viral infections by limiting antigen sensitivity of CD8^+^ T cells and restricting activation [134]. It remains unknown and intriguing how this function is regulated metabolically.

The anti-inflammatory cytokine IL-10 affects two major metabolic pathways in IL-10-receptive macrophages: the oxidative phosphorylation (OXPHOS)/glycolysis balance and sphingolipid metabolism. (A) IL-10 induces DNA damage inducible transcript 4 (DDIT4) via activation (phosphorylation) of signal transducer and activator of transcription 3 (STAT3), which inhibits the mammalian target of rapamycin complex 1 (mTORC1). This in turn induces the switch from glycolysis to OXPHOS and inhibits glucose transporter 1 (GLUT-1) translocation to the plasma membrane, while reducing pro-inflammatory hypoxia-inducible factor 1-alpha (HIF-1α) and IL-1β. (B) IL-10 also inhibits saturated very long chain (VLC) ceramide (Cer24:0) production via ceramide synthase (CerS2) in the ER. Cer24:0 induces pro-inflammatory gene expression via transcription factor c-REL, as opposed to long chain ceramides (Cer16:0). IL-10 therefore limits pro-inflammatory gene expression by rewiring sphingolipid metabolism. ER: Endoplasmic reticulum.

## Metabolic regulation of trained immunity

Trained immunity describes the epigenetic and transcriptional reprogramming of bone marrow-derived myeloid cells following exposure to non-lethal microbial stimuli [135]. It engenders broad protection against subsequent infection, as opposed to tolerance in which repeated exposure leads to suppressed inflammatory cell phenotypes [136]. Despite clear definitions of trained immunity, especially upon vaccination with BCG [136, 137], controversy remains in the field whether some protective effects may be attributed to local tissue remodeling (i.e. in the lung) instead, or in addition to myelopoietic training [138]. The elicitation of trained immunity depends in part on IL-1β and type 1 IFN signaling [139, 140] acting at the level of progenitors in the bone marrow [136]. However, the exact role of cell-extrinsic cytokine signaling on myeloid progenitors that results in their phenotypic and functional changes, remains largely unclear. Along with epigenetic and transcriptional changes, trained immunity is linked to adaptations in the metabolic activity of “trained” myeloid cells [141–143]. Thus, manipulation of trained immunity (e.g., exogenously by administration of defined microbial products) is a potential way to influence the outcomes of inflammation and infection. Accordingly, considerable efforts are being made to understand the basic mechanisms of establishing trained immunity, its consequences to myeloid cell activity and effects on the physiology of different organs in disease. In our view, investigating trained immunity with the new immunometabolic techniques outlined here is ideal to link phenotype with function, as discussed below.

Trained immunity can be provoked in different myeloid populations including monocytes, neutrophils and likely any cell type that develops from erythroid-myeloid progenitors or other stem cells in the bone marrow. A hallmark characteristic of “trained” myeloid cells that exit the bone marrow are changes in metabolism [144–146]: Monocytes that exit the bone marrow following beta-glucan injection (i.e., a fungal cell wall component) or mycobacterial infection (via Bacillus Calmette-Guérin; BCG) [147] have increased glycolysis, OXPHOS and mTORC1 activity [142, 148]. These metabolic changes are coincident with widespread epigenetic reprogramming that follows the first stimulus, which was the foundational observation that established the trained immunity field [135, 146]. Thus, increased central metabolism and epigenetic changes are the two characteristics that define trained myeloid cells. A common explanation for increased central metabolism following training is that more acetyl-CoA is needed for histone modifications. However, precise measurement of acetyl-CoA amounts in individual trained cells across time is lacking. Therefore, a key question is whether ongoing generation of acetyl-CoA is sufficient to explain the observed changes in glycolysis and TCA cycle activity. Other metabolic pathways have been suggested to control trained immunity such as mevalonate production to contribute to cholesterol production [149]. However, the metabolic changes thus far reported cannot explain why these changes are needed. Two important questions come to the fore about changes in metabolism following training: (1) Given that trained myeloid cells that exit the bone marrow are post-mitotic, changes in central energy pathways are unnecessary for cell division. Do they serve another purpose? (2) How can we devise an experimental approach to link changes in central metabolism to trained myeloid cell function, and thereby identify both reasons for metabolic and functional changes in trained cells? In our view, addressing these questions can provide an important avenue in manipulating innate immune training such that trained cells can develop enhanced functions or survival in e.g., infections. By contrast, a reasonable expectation about innate immune training is that a similar process alters bone marrow hematopoiesis in chronic cancer, eliciting increased output of myeloid-derived suppressor cells (MDSCs) [150], which also have widespread changes in metabolic pathways and may be “cancer trained” versions of myeloid populations observed in conventional training experiments [151].

To obtain new information about metabolic changes in trained immunity, access to the cells via the blood and bone marrow is an advantage. Application of flow cytometry based single-cell metabolic profiling (SCENITH) [152] and staining for dividing cells within trained versus non-trained myeloid cells is the first step and would enable us to gain information about ATP consumption in dividing and non-dividing populations. Then, single cells (or at least small numbers of similar cells) can be used for untargeted metabolomics and proteomics. The necessary “positive control” could be fulfilled by single cell ATAC-seq as epigenetic changes indicate the occurring training process at the epigenetic level. These steps would likely reveal the expected changes in central metabolism such as increased mevalonate production and increased TCA cycle activity, but may point to proteomic distinctions that would give clues to other pathways changed during training.

## Metabolomic biomarkers of inflammatory disease

**Fig. 3:**
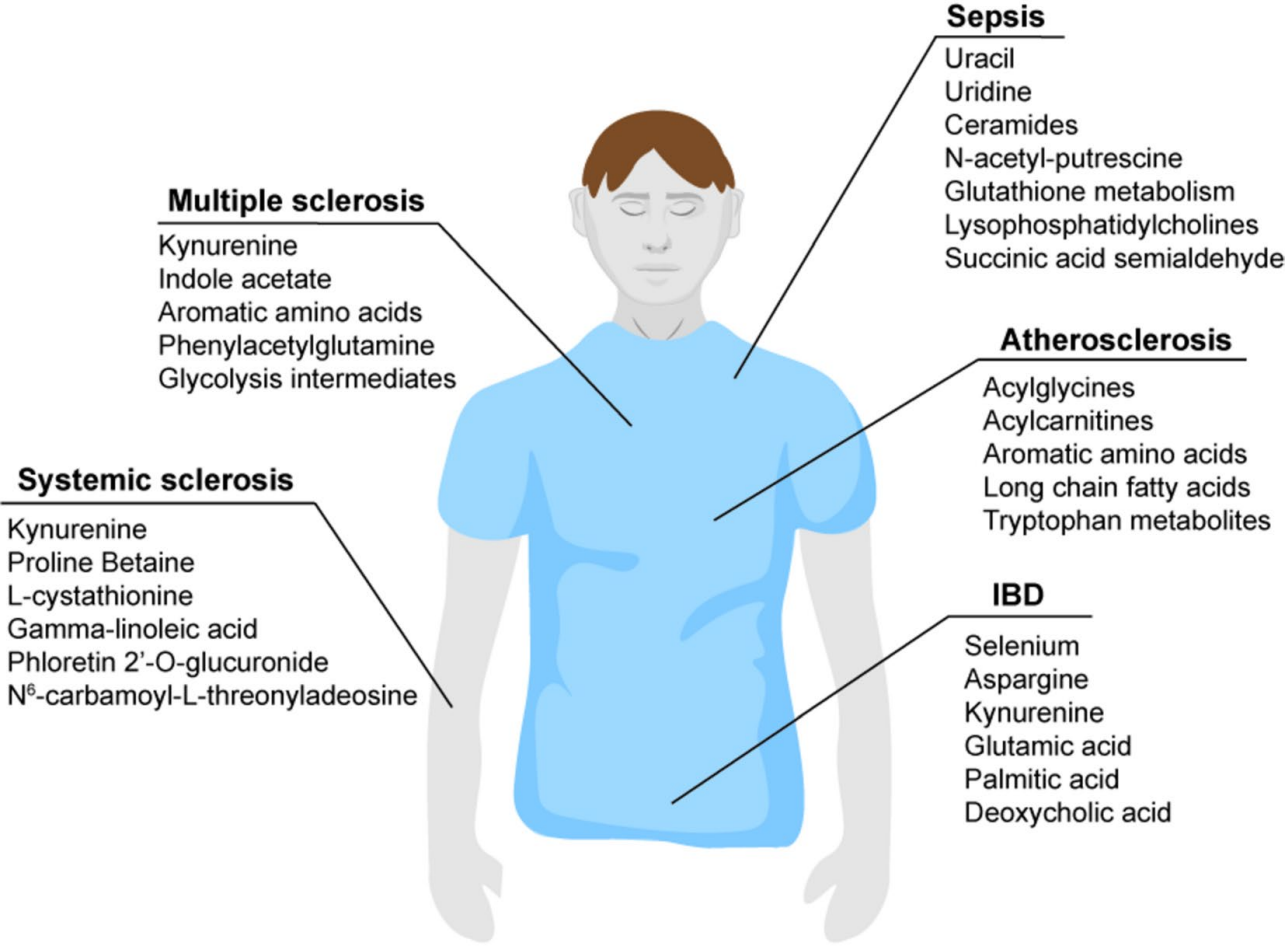
Newly suggested potential metabolomic biomarkers for inflammatory disease

Metabolomic analysis has become not only essential for drug development [153] and understanding modes of action– such as the recent metabolomic profiling of TCA cycle-derived metabolites in macrophages upon glucocorticoid (GC) treatment, which identified itaconate as a driver of anti-inflammatory effects of GC treatment [154]. In addition, metabolomic biomarkers may be useful for diagnosis and stratification of inflammatory diseases using non-invasive biological samples (i.e. blood, urine), despite challenges such as unstable metabolites. In this section we discuss recently discovered metabolomic biomarkers suggested to be useful in understanding complex inflammatory diseases (Figure 3).

### Systemic sclerosis

Systemic sclerosis (Ssc) is an autoimmune rheumatic disease, which can affect different organs (including the lung, leading to development of interstitial lung disease; ILD). It is grouped into limited Ssc affecting the extremities and face and diffuse Ssc, which also affects arms, legs and trunk [155]. Metabolomic plasma/serum analysis could aid in identifying patients at risk of developing diffuse Ssc and interstitial lung disease. Plasma targeted and untargeted mass spectrometric analysis of Ssc patients and healthy controls (HC) found increased activity of the kynurenine pathway in Ssc patients (Ssc n = 58; HC n = 48) [104]. This is in line with a previous untargeted study analyzing plasma (Ssc n = 58; HC n = 28), which suggested kynurenine as a potential biomarker to differentiate between Ssc patients with and without ILD [105]. In contrast, a more recent, larger untargeted metabolomic serum study (Ssc n = 127; HC n = 142) proposed a different metabolite panel to identify patients at risk of developing Ssc-ILD, including proline betaine, phloretin 2’-O-glucuronide, gamma-linoleic acid and L-cystathionine [156]. Kynurenine and unsaturated fatty acid metabolism were also identified by untargeted LC-MS analysis of plasma to differentiate patients with different systemic autoimmune diseases (SAD), while a byproduct of spermidine, N^6^-carbamoyl-l-threonyladenosine, was decreased across SAD patients compared to healthy controls (SAD patients *n* = 228; HC *n* = 56) [106]. In conclusion, the potential biomarker kynurenine, which has been identified as strongly altered in SSc patients in several studies, will need to be confirmed in larger studies, together with the metabolite panel consisting of proline betaine, phloretin 2’-O-glucuronide, gamma-linoleic acid and L-cystathionine.

### Multiple sclerosis

Multiple sclerosis (MS) is the most common neurological disease in young adults. Distinct courses of disease progression include relapsing-remitting MS (RRMS) marked by intermittent periods of neurological stability and relapsing episodes and secondary progressive MS (SPMS), where neurological function declines steadily over time [157, 158]. Conceivably, metabolomic biomarkers in plasma or serum could aid in the differentiation of active/inactive MS and progressive/non-progressive MS as well as identifying ongoing neurological inflammation. Untargeted combined GC-MS and LC-MS metabolomic serum/plasma analysis of a large cohort (MS patients *n* = 637; HC *n* = 317) identified abnormalities in aromatic amino acid metabolism, increased production of microbial derived metabotoxins (indole acetate, phenylacetylglutamine) and decreased lactate metabolites in patients with MS compared to healthy controls. Kynurenine/tryptophan metabolism was reduced and associated with a higher disability score, suggesting kynurenine as a potential stratifying biomarker in MS [159]. Another, more recent untargeted serum analysis comparing RRMS patients and HC (RRMS *n* = 25; HC *n* = 14) reported clear separation of the two groups based on serum glycolytic metabolites [160]. In particular the large cohort untargeted metabolomic study, which linked reduced kynurenine to an increased disability score is promising, but further studies including MS subtypes (i.e. active/non-active, progressive/non-progressive) will be necessary to further validate kynurenine as a metabolic biomarker for multiple sclerosis.

### Atherosclerosis

Atherosclerosis is an arterial inflammatory disease and major cause for cardiovascular disease (CVD) and stroke. Lipid accumulation in the arteries leads to formation of atherosclerotic lesions and plaques, fueled by a chronic inflammatory milieu [161, 162]. Plaques can be differentiated between stable and unstable, which are prone to rupture, resulting in arterial obstruction. Biomarkers exist to identify the risk of developing CVD, such as high sensitivity C-reactive protein (hsCRP), which is used as an inflammatory marker to identify atherosclerotic risk and to determine anti-inflammatory effects of statin therapy [163, 164]. To differentiate between stable and unstable plaques and underlying metabolic pathways, a recent study analyzed spatially resolved metabolites using MALDI-MSI in atherosclerotic plaques and integrated these with RNAseq data. Despite the small sample size (*n* = 5 stable, *n* = 4 unstable plaques), this approach identified long chain fatty acids, acylcarnitines and acylglycines enriched in stable plaques, while unstable plaques were marked by an accumulation of ROS, tryptophan metabolites and aromatic amino acids [165]. It will be essential to further validate these findings in a large cohort study, to enable metabolic biomarker analysis of atherosclerotic plaques.

### Inflammatory bowel diseases

Inflammatory bowel diseases (IBD) are chronic inflammatory disorders of the gastrointestinal (GI) tract and affect approximately 0.3% of the population in Europe and North America [166]. The two most common IBDs, initiated by dysregulated mucosal immune responses and host-microbe interaction, are Ulcerative Colitis (UC) and Crohn’s Disease (CD). UC, as opposed to CD, is limited to the colon. Metabolomic analysis of serum and colonic biopsies may aid in diagnosing and stratifying IBD patient groups. Using an untargeted combined metabolomic method (GC-MS and UHPLC-MS), a small study (Active UC *n* = 18; Remission UC *n* = 10; HC *n* = 14) identified highly increased kynurenine, asparagine, and glutamic acid in active UC, as well as linoleic acid metabolism [107]. In contrast, untargeted serum metabolomics of CD patients and HC (CD *n* = 158; HC *n* = 158) suggested deoxycholic acid and palmitic amide as biomarkers for CD [167], while metabolomic analysis of IBD colonic tissue (CD *n* = 32, UC *n* = 32, HC *n* = 30) identified reduced selenium as a distinct feature of CD colonic biopsy samples [168]. In addition to using selenium as a biomarker to stratify CD and UC patients, this study proposed selenium supplementation as treatment strategy in CD patients, since it discovered selenium-dependent anti-inflammatory T cell responses [168]. While these results are promising in their distinct metabolic features of Ulcerative Colitis and Crohn’s Disease, large cohort studies will be necessary to validate kynurenine, asparagine and glutamic acid as well as deoxycholic acid, palmitic amide and selenium as biomarkers.

### Sepsis

Sepsis results from dysfunctional immune responses to infection, leading to life threatening inflammation and organ dysfunction. Sepsis is complex and may be caused by a range of different pathogens. In addition to blood biomarkers, metabolomic analysis of serum/ plasma could help to identify trauma patients or those with inflammation at risk of developing sepsis, or identify microbial metabolites contributing to blood stream infections. Prior studies identified lipid metabolism, particularly ceramides and lysophosphatidylcholines decreased in sepsis [169, 170], although association with disease severity is controverse [170, 171]. Untargeted metabolomic analysis was performed in patients with trauma and trauma/sepsis (*n* = 30 each), upon which three biomarkers were suggested to identify trauma patients with sepsis risk: succinic acid semialdehyde, uracil and uridine [172]. In contrast, untargeted LC-MS of serum with parallel transcriptomic analysis found distinct glutathione metabolism between patients with sepsis and systemic inflammatory responses (Sepsis *n* = 16; SIRS *n* = 11) [173]. Untargeted LC-MS analysis of polymorphonuclear neutrophils, of which trafficking and function is dysregulated during sepsis [174], found decreased lactate in sepsis compared to non-septic patients (Sepsis *n* = 14, non-septic acute appendicitis *n* = 26, HC *n* = 19) [175]. This study furthermore linked inhibited neutrophil glycolysis to immune dysfunction. Most recently, metabolomic analysis of plasma from gram-negative blood stream infected (BSI) patients, including *Escherichia Coli*, *Klebsiella pneumoniae* and *Pseudomonas aeruginosa*, (BSI *n* = 21; HC *n* = 21) identified bacterially derived acetylated polyamines, specifically putrescine derived N-acetylputrescine as a metabolic marker for BSI [171]. Targeting this pathway by inhibiting polyamine acetyltransferases increases intracellular antibiotic accumulation and resensitizes antimicrobial resistant bacteria. In conclusion, proposed metabolomic biomarkers for sepsis are as diverse as sepsis is complex. All studies discussed here are small cohort studies and proposed biomarkers to identify trauma or systemic inflammation patients with sepsis risk, or gram-negative blood stream infections will need to be validated in larger cohorts.

### Summary of metabolomic biomarkers of inflammatory disease

Untargeted metabolomic analysis of blood/biopsy samples may aid to diagnose or stratify complex inflammatory diseases such as systemic sclerosis, multiple sclerosis, atherosclerosis, IBD and sepsis. While distinct metabolites were discussed as metabolomic biomarkers for these (Figure 3), one pathway stands out since it is altered across the board in systemic sclerosis, multiple sclerosis, atherosclerosis and IBD: The tryptophan/kynurenine pathway. Kynurenine has been implicated in pathophysiologies ranging from inflammatory disease to depression [176, 177], while also supporting tumor growth as substrate for NAD^+^ biosynthesis [178], 1-carbon metabolism and as intermediate for anti-ferroptotic kynurenine-metabolites [36]. Confirming metabolites of the tryptophan/kynurenine pathway in large cohort studies as metabolomic biomarkers to stratify systemic sclerosis, multiple sclerosis or IBD subtypes and progression or to identify unstable atherosclerotic plaques would be valuable for diagnosis as well as understanding susceptibility for these inflammatory disorders. Since the advancing LC-MS technology comes with reduced cost for (targeted) metabolomic measurements, clinical implementation of potential metabolomic biomarkers for disease diagnosis and stratification could be feasible from a cost perspective, although implementation in low- and middle-income countries as well as the establishment of reliable metabolite standards remain as challenges.

Non-targeted metabolomic analysis of serum/plasma and biopsy samples identified altered metabolites with potential biomarker function to diagnose and stratify inflammatory diseases like systemic sclerosis, multiple sclerosis, atherosclerosis, inflammatory bowel disease (IBD) and sepsis [104–107, 156, 159, 160, 165, 167, 168, 171–173].

## Conclusion and perspectives

Progression from inflammation to resolution, shaped by efferocytosis and immunosuppressive cytokines TGF-β and IL-10 is critical to prevent development of chronic inflammation. Metabolic programs such as glycolysis and fatty acid metabolism, but importantly also the tryptophan/kynurenine pathway as well as polyamines are key nodes and potential targets of macrophage driven inflammation resolution. Particularly kynurenine was identified as essential for efferocytosis and inflammation resolution as well as a biomarker in diverse inflammatory conditions. Spatial and single cell metabolomic analysis, integrated with transcriptomic and proteomic data as well as CRISPR screens will be necessary to fully understand SLC transport of metabolites, amino acids and lipids during efferocytosis and chronic inflammatory disease. The next challenge of metabolomic analysis in inflammation will be not only to further improve reproducibility and analysis methods to identify unknown metabolites, but also to integrate metabolomic, proteomic and transcriptomic data, at single cell level and with spatial resolution. This will require new bioinformatic deep learning tools [179] and will result in unprecedented rich data providing functional (metabolite), regulatory (enzymes and transporters) and underlying transcriptomic information at the same time; ideally over time and with spatial single cell or subcellular resolution of ex vivo, in vitro and in vivo tissue sites. Combining single cell and spatial metabolomics with other single cell metabolic profiling approaches such as Met-flow and SCENITH, identifying metabolite transporter expression at single cell level by flow cytometry and linking protein synthesis to metabolic inhibition [152, 180], will provide a more holistic metabolomic picture beyond metabolite expression.

## Data Availability

No datasets were generated or analyzed for this manuscript.
